# Supplementary material for: The effect of cycling hypoxia on MCF-7 cancer stem cells and the impact of their microenvironment on angiogenesis using human umbilical vein endothelial cells (HUVECs) as a model
Source: PeerJ. 2019 Jan 8;7:e5990. doi: 10.7717/peerj.5990 (PMC6361090; doi:10.7717/peerj.5990)
Supplement: Supplemental Information 1 — The raw data of identification CD44+/CD24- phenotype content by flow cytometry. [file peerj-07-5990-s001.docx]

***Identification of CD44^+^/CD24^-^ Phenotype Content by Flow Cytometry***

parent MCF-7 cells (1.1%) parent MCF-7 cells (1.09%) parent MCF-7 cells (0.8%)


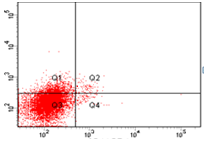

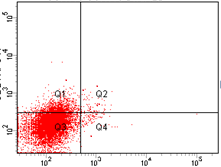

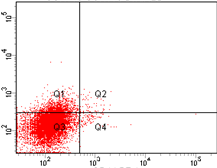


**Group 1**

unsorted (33.1%) unsorted (36.3%) unsorted (30.1%)


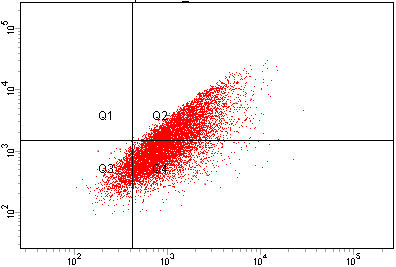

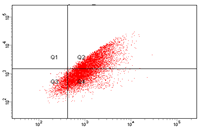

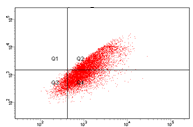


**Group 2**

After 3 days of sorting After 3 days of sorting After 3 days of sorting

(75.5%) (84.4%) (82.1%)


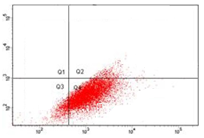

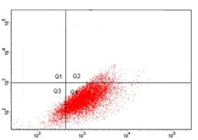

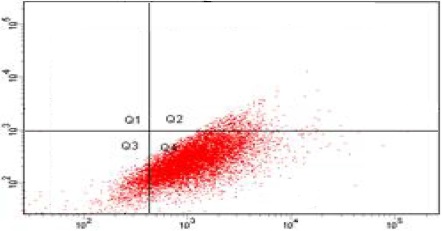


**Group 3**

CSCs after 21 days of sorting CSCs after 21 days of sorting CSCs after 21 days of sorting

(31.6%) (39.4%) (35.5%)


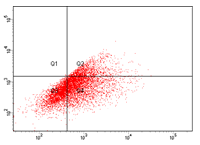

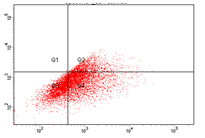

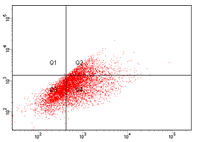


**Group 4**

Surface marker CD44+/CD24- expression analyzed for: **(Group 1 triplicate)** parent MCF-7 cells (1.0%), **(Group 2 triplicate)** unsorted mammospheres (33.2%); **(Group 3 triplicate)** sorted CSCs mammospheres after 3 days of sorting (81.0%); **(Group 4 triplicate)** CSCs mammospheres after 21 days of sorting (35.5%)

**INTR.20** (34.6%) **INTR.20** (44.2%) **INTR.20** (40.5%)


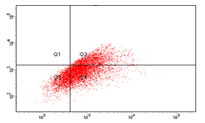

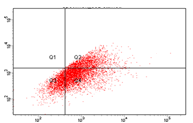

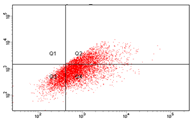


**Group 5**

**CONT.5** (45.6%)  **CONT.5** (57.7%) **CONT.5** (51.6%)


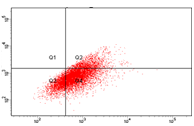

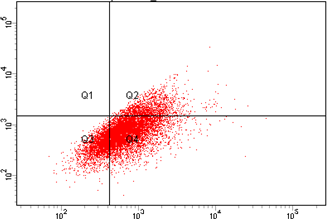

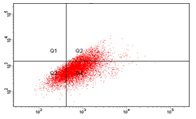


**Group 6**

**INTR.40** (0.30%)  **INTR.40** (0.32%)  **INTR.40** (0.28%)


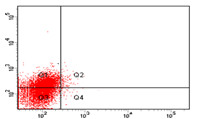

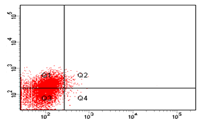

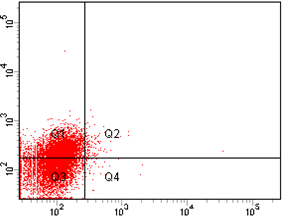


**Group 7**

**CONT.15** (0.46%) **CONT.15** (0.54%) **CONT.15** (0.5%).


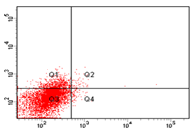

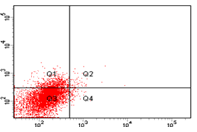

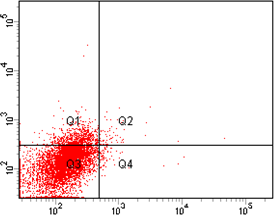


**Group 8**

Surface marker CD44+/CD24- expression analyzed for: **(Group 5 triplicate)** hypoxic CSCs mammospheres **INTR.20** (39.8%); **(Group 6 triplicate)** hypoxic CSCs mammospheres **CONT.5** (51.6%); **(Group 7 triplicate)** hypoxic CSCs mammospheres **INTR.40** (0.3%); **(Group 8 triplicate)** for hypoxic CSCs mammospheres **CONT.15** (0.5%).
