# Supplementary figures and images for: The effect of cycling hypoxia on MCF-7 cancer stem cells and the impact of their microenvironment on angiogenesis using human umbilical vein endothelial cells (HUVECs) as a model
Source: PeerJ. 2019 Jan 8;7:e5990. doi: 10.7717/peerj.5990 (PMC6361090; doi:10.7717/peerj.5990)

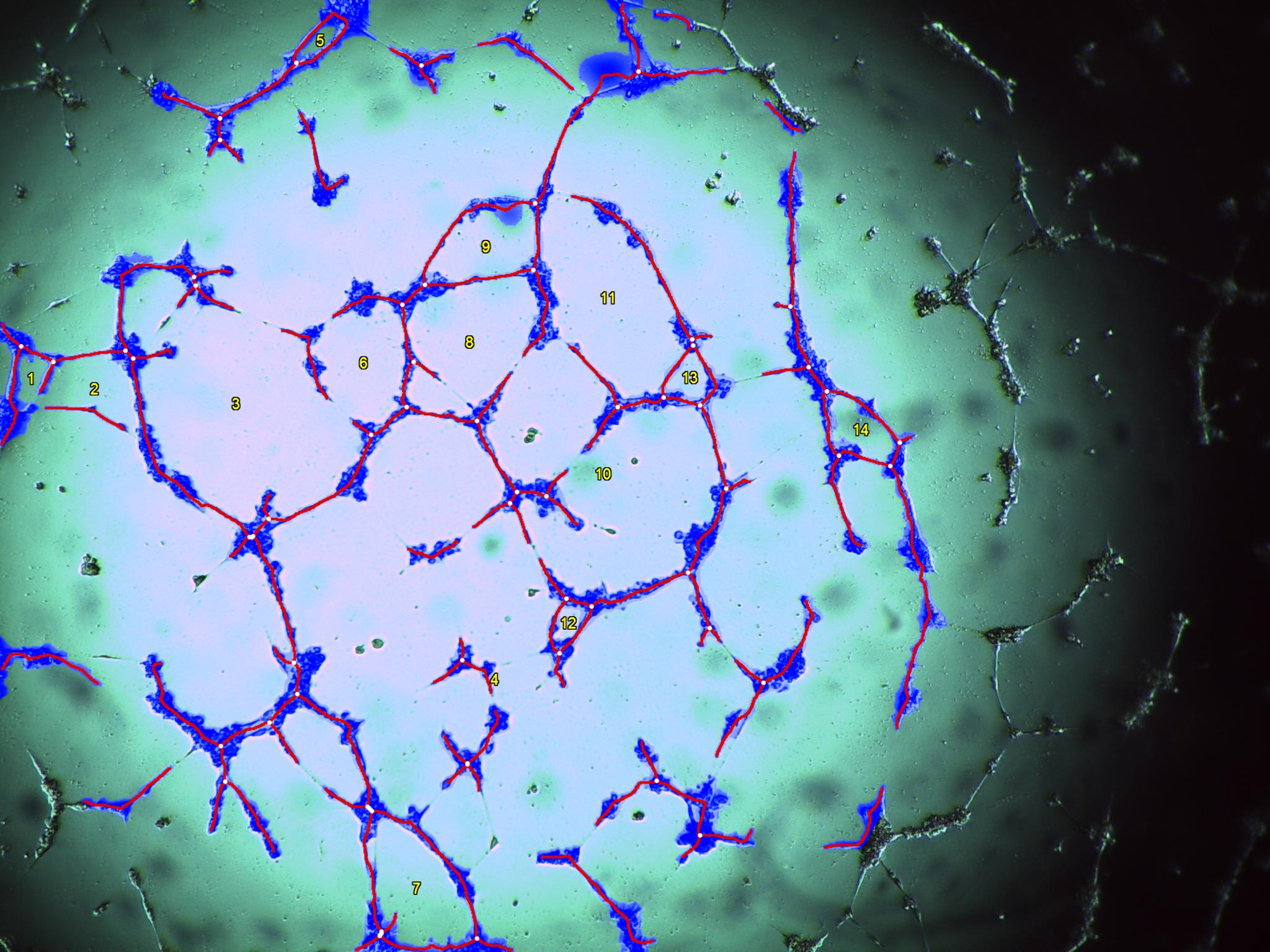

Supplement: Supplemental Information 9 — Assessment of capillary-like tubular structure in the control. [file peerj-07-5990-s009.zip › controlExp01.jpg]

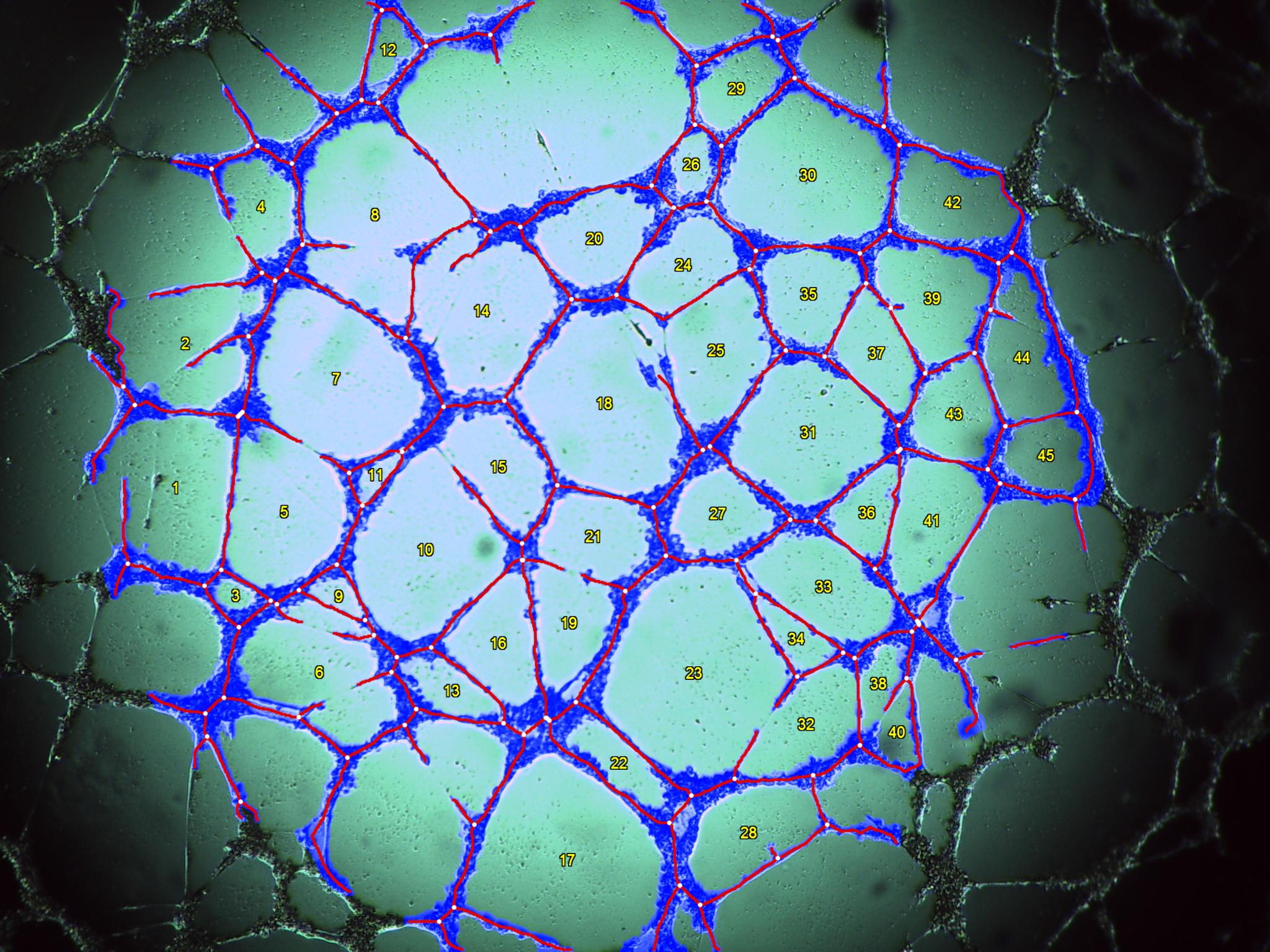

Supplement: Supplemental Information 10 — Assessment of Capillary-likeTubular Structure in INTR.10 [file peerj-07-5990-s010.zip › an_int10.jpg]

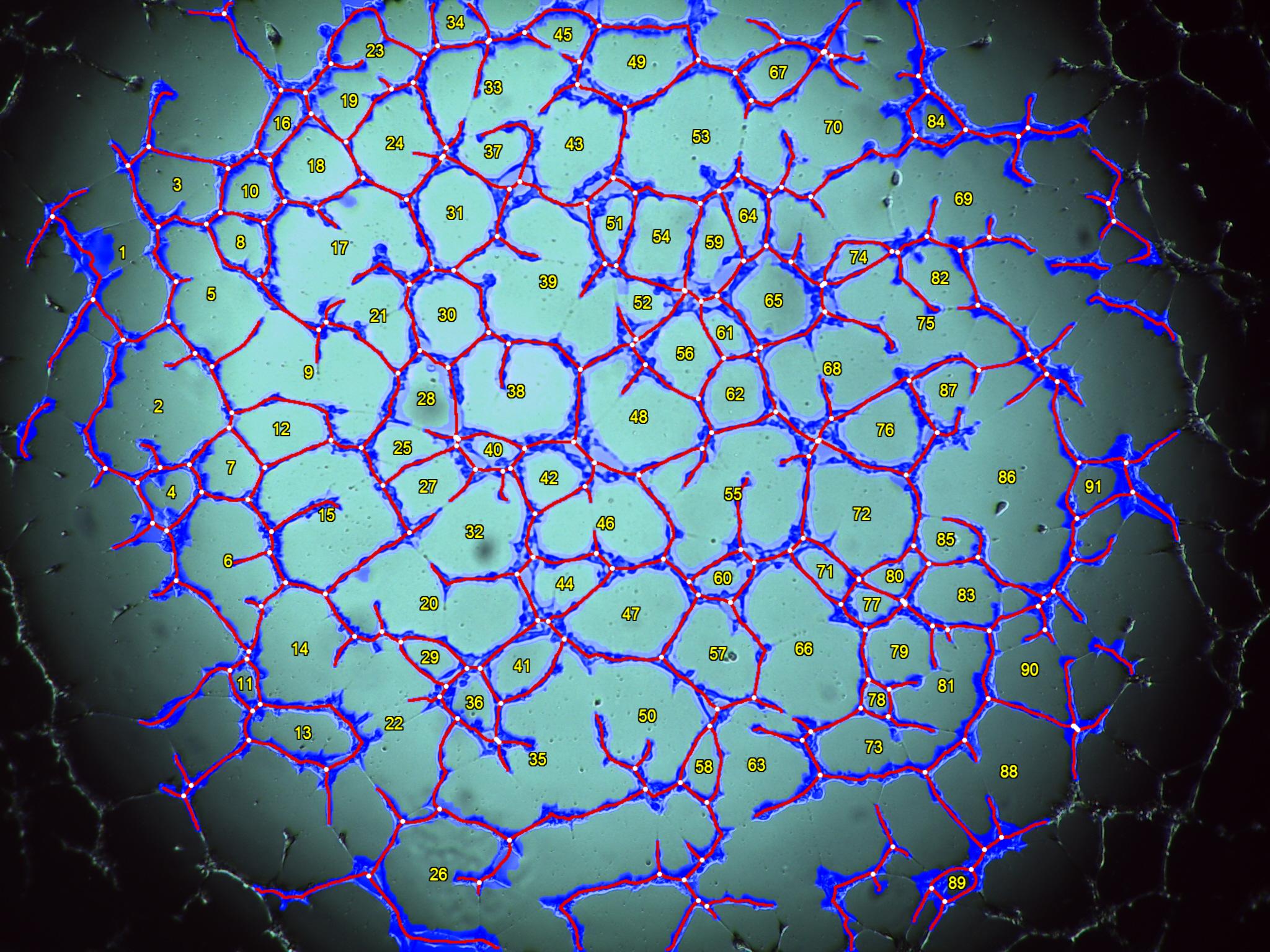

Supplement: Supplemental Information 11 — Assessment of capillary-like tubular structure formation inINTR.20. [file peerj-07-5990-s011.zip › int.20.jpg]

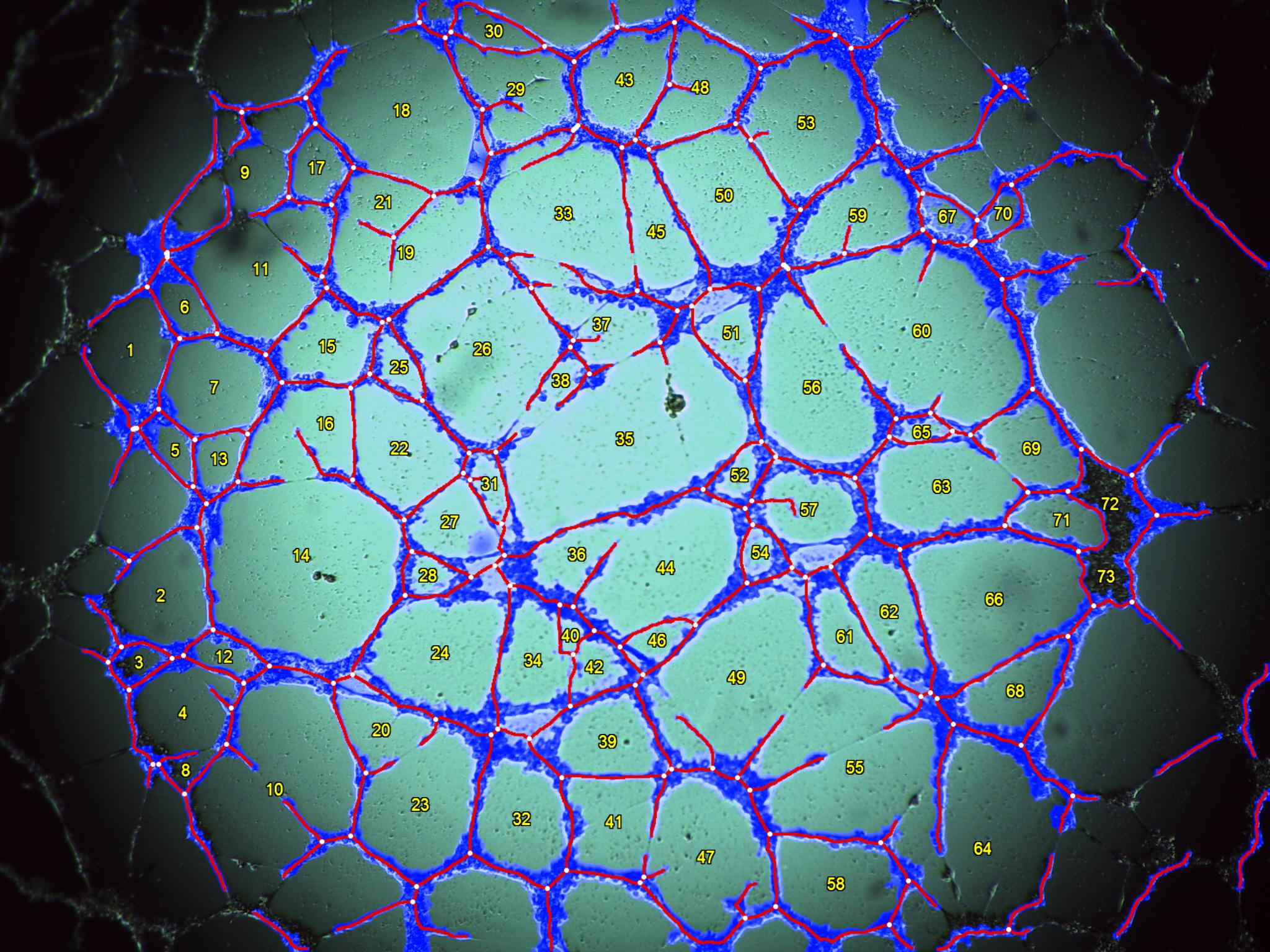

Supplement: Supplemental Information 12 — Assessment of capillary-like tubular structure formation in INTR.30. [file peerj-07-5990-s012.zip › an_int30.jpg]

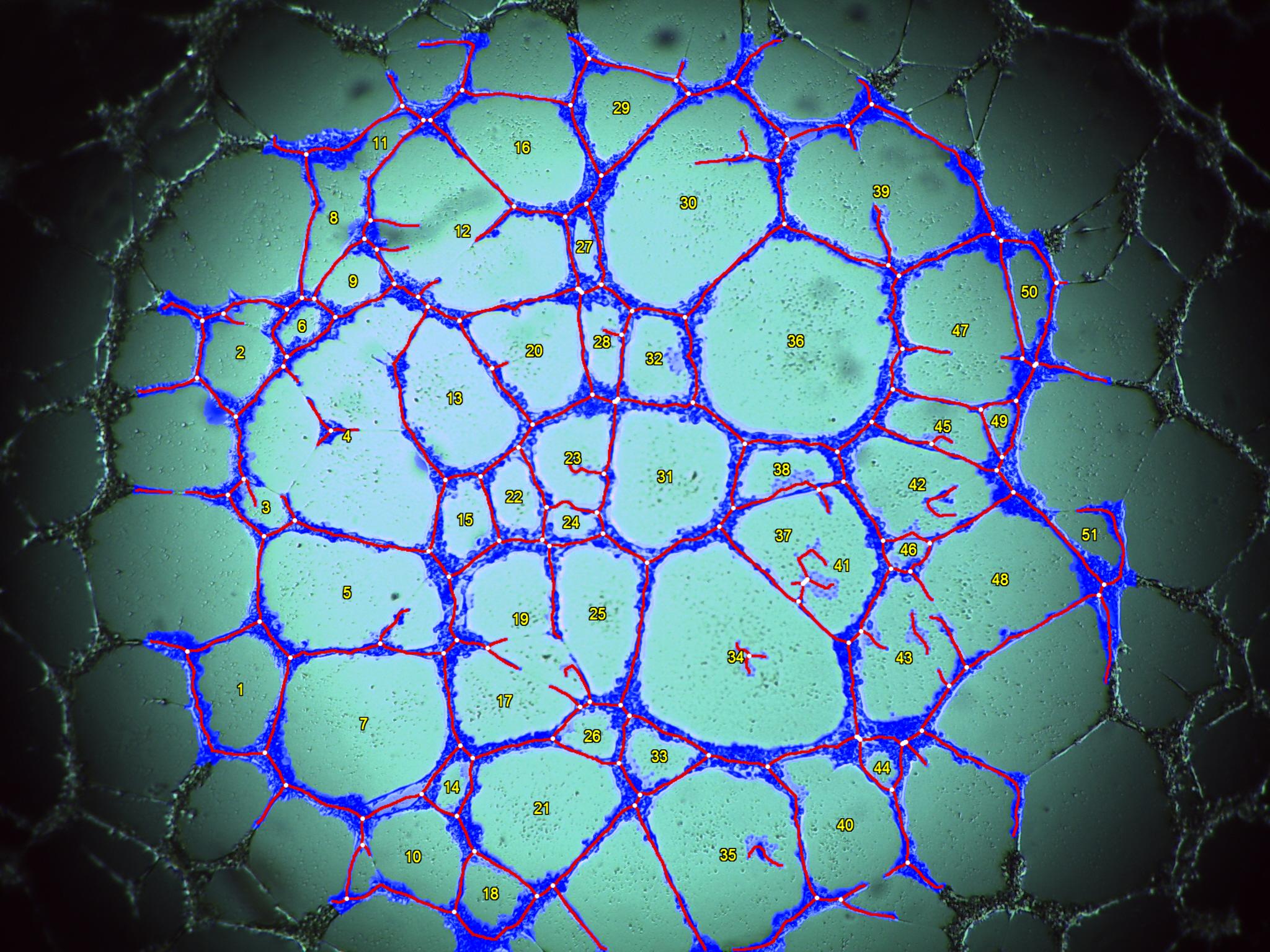

Supplement: Supplemental Information 13 — Assessment of capillary-like tubular structure in INTR.40. [file peerj-07-5990-s013.zip › INT40.jpg]

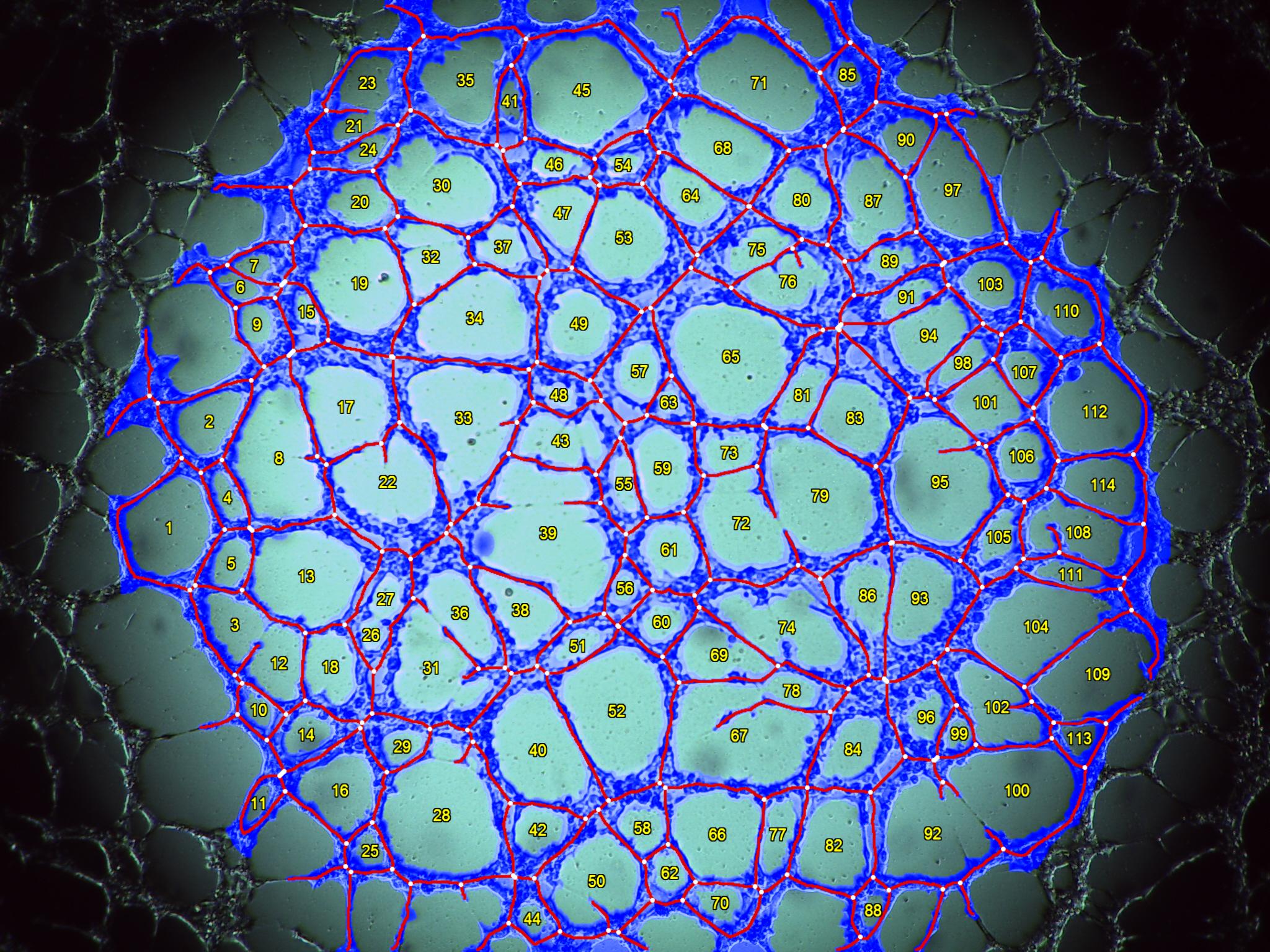

Supplement: Supplemental Information 14 — Assessment of capillary-like tubular structure formation CONT.5. [file peerj-07-5990-s014.zip › CONT.5.jpg]

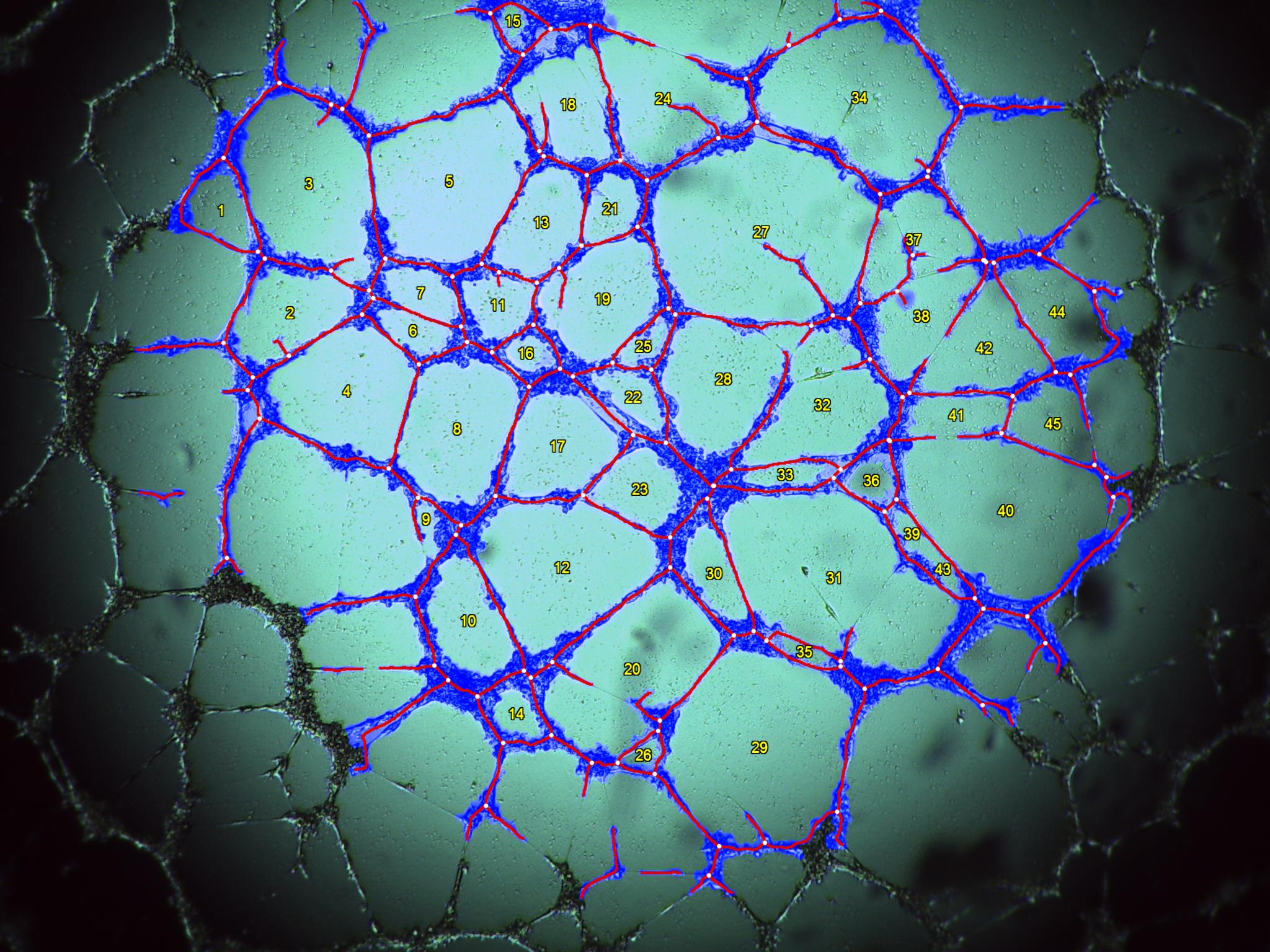

Supplement: Supplemental Information 15 — Assessment of capillary-like tubular structure formation in CONT.10. [file peerj-07-5990-s015.zip › cont.10.jpg]

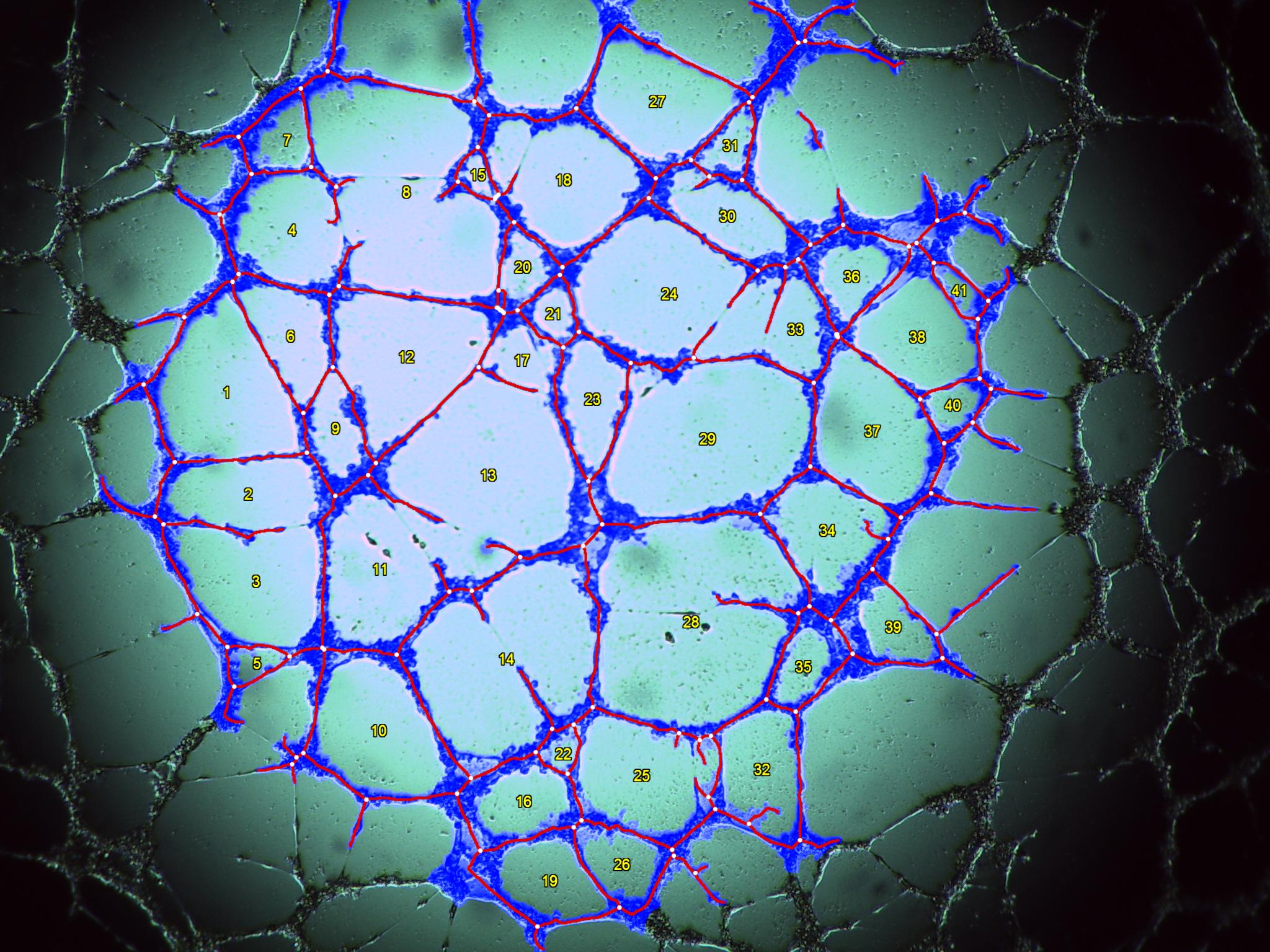

Supplement: Supplemental Information 16 — Assessment of capillary-like tubular structure formation in CONT.15. [file peerj-07-5990-s016.zip › an_con15_10h_Exp01.jpg]
